# A Novel Matrine Derivative WM130 Inhibits Activation of Hepatic Stellate Cells and Attenuates Dimethylnitrosamine-Induced Liver Fibrosis in Rats

**DOI:** 10.1155/2015/203978

**Published:** 2015-06-18

**Authors:** Yang Xu, Zhangxiao Peng, Weidan Ji, Xiang Li, Xuejing Lin, Liqiang Qian, Xiaoya Li, Xiaoyun Chai, Qiuye Wu, Quangen Gao, Changqing Su

**Affiliations:** ^1^Department of Molecular Oncology, Eastern Hepatobiliary Surgery Hospital and National Center of Liver Cancer, Second Military Medical University, Shanghai 200438, China; ^2^Deparment of Pharmacy, Second Military Medical University, Shanghai 200433, China; ^3^Department of General Surgery, Wujiang No. 1 People's Hospital, Suzhou 215200, China

## Abstract

Activation of hepatic stellate cells (HSCs) is a critical event in process of hepatic fibrogenesis and cirrhosis. Matrine, the active ingredient of *Sophora*, had been used for clinical treatment of acute/chronic liver disease. However, its potency was low. We prepared a high potency and low toxicity matrine derivate, WM130 (C_30_N_4_H_40_SO_5_F), which exhibited better pharmacological activities on antihepatic fibrosis. This study demonstrated that WM130 results in a decreased proliferative activity of HSC-T6 cells, with the half inhibitory concentration (IC_50_) of 68 *μ*M. WM130 can inhibit the migration and induce apoptosis in HSC-T6 cells at both concentrations of 68 *μ*M (IC_50_) and 34 *μ*M (half IC_50_). The expression of *α*-SMA, Collagen I, Collagen III, and TGF-*β*1 could be downregulated, and the protein phosphorylation levels of EGFR, AKT, ERK, Smad, and Raf (p-EGFR, p-AKT, p-ERK, p-Smad, and p-Raf) were also decreased by WM130. On the DMN-induced rat liver fibrosis model, WM130 can effectively reduce the TGF-*β*1, AKT, *α*-SMA, and p-ERK levels, decrease the extracellular matrix (ECM) formation, and inhibit rat liver fibrosis progression. In conclusion, this study demonstrated that WM130 can significantly inhibit the activation of HSC-T6 cells and block the rat liver fibrosis progression by inducing apoptosis, suppressing the deposition of ECM, and inhibiting TGF-*β*/Smad and Ras/ERK pathways.

## 1. Introduction

Liver fibrosis, a common pathological outcome of numerous chronic liver diseases, is characterized by intrahepatic connective tissue proliferation which results in a diffuse and excessive deposition of extracellular matrix (ECM) in the liver [1]. Advanced fibrosis can lead to hepatic lobular reconstruction and formation of fibrous septa and pseudolobules or nodules and then develop into liver cirrhosis, which is a kind of serious and incurable liver disease. Therefore, attenuating or reversing hepatic fibrosis is a crucial strategy for preventing liver cirrhosis. Currently, two main strategies are used for hepatic fibrosis treatment, eliminating pathogenic factors such as antivirus treatment and abstinence of drinking and decreasing synthesis and deposition of ECM in liver tissues. However, glucocorticoids, widely used as treatment of autoimmune or alcoholic liver diseases in clinic, have many adverse reactions; silymarin and other drugs, used as hepatic protective agents in patients with liver disease, still need large randomized controlled clinical studies to determine whether they can decrease the incidence of hepatic fibrosis and improve patients' prognosis with long-term use. Currently, there are few approaches for treatment of liver fibrosis, and it is quite necessary to find and study some novel antifibrotic drugs.

Activation of hepatic stellate cells (HSCs) is acknowledged as a critical event in process of hepatic fibrosis [2]. Hepatocyte damage caused by a variety of pathogenic factors can lead to the high expression of growth-stimulating factors, such as transforming growth factor-*β* (TGF-*β*), platelet-derived growth factor (PDGF), endothelin (ET), fibroblast growth factor (FGF), connective tissue growth factor (CTGF), and leptin [3]. These growth factors are released from hepatocytes and induce the activation of HSCs through TGF-*β*/Smad pathway [4–8] and Ras/ERK [9–11] pathway. The quiescent HSCs transdifferentiate into fibrogenic, proliferative, and contractile myofibroblast-like phenotypes, as represented by the expression of *α*-smooth muscle actin (*α*-SMA) [12], the production of excessive collagens, and the enhanced proliferation and migration activities. Therefore, inhibiting activation of HSCs is considered as the most effective strategy for prevention or treatment of liver fibrosis.

**Figure 1:**
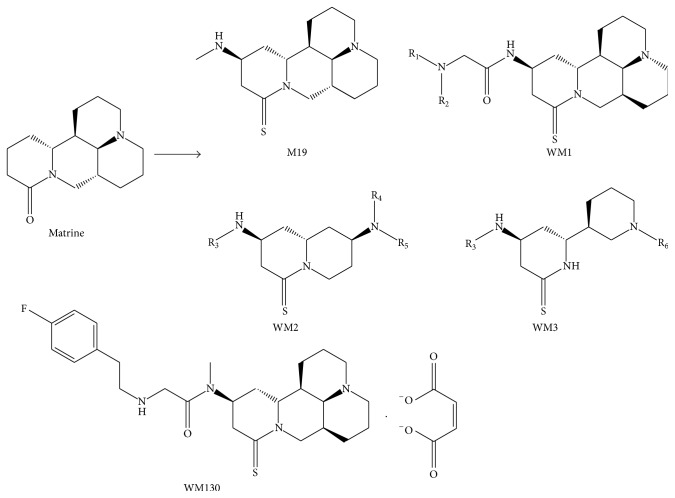
Chemical structures of matrine and its derivatives, including M19 (C_16_N_3_H_27_S), and compound series. M19 was obtained through thiosulfate and side chain Michael addition, and the series of WM1, WM2, and WM3 were those containing an alkyl group or alkyl groups in place of one or more hydrogen atoms on M19. WM130 was obtained through reconstructing the structure of M19 with 18-methylamino-acylated and amino side chain addition.

Matrine and oxymatrine, the main active ingredients of* Sophora flavescens* (*S. flavescens* Ait), possess a variety of biological activities, including anti-inflammatory, immunosuppressive, antitumor, and anti-liver fibrosis [13–16], and had been used for clinical treatment of acute/chronic liver diseases. In addition, it was reported that matrine can attenuate lipopolysaccharides/D-galactosamine-induced acute liver injury in mice and CCl_4_-induced hepatic fibrosis in rats [17, 18]. However, the potency of matrine and oxymatrine was low. Oxymatrine could be rapidly eliminated because of its short half-life (*t*_1/2_ = 0.5 h) [19]. Natural products are an important source of novel lead structures for the synthetic and combinatorial chemistry aspects of new drug discovery. So it was quite interesting for us to prepare high potency and low toxicity matrine derivates. It is confirmed that after replacing carbonyl oxygen atom by sulfur atom, the pharmacological activity of matrine derivates has been greatly increased. From structural modification, we have obtained more than 40 compounds (WM1 series, WM2 series, and WM3 series) with different alkyl replacing, in which the semisynthetic derivative of matrine M19 (C_16_N_3_H_27_S) had better pharmacological activities and lower side effect or toxicity than matrine and its other derivatives [20]. M19 can inhibit DMN-induced or bile duct ligation- (BDL-) induced liver fibrosis [21] and liver cancer by blocking AKT and EGFR signaling pathways. However, M19 is unstable because of its tertiary amine group. M19 can easily turn back into thiosulfate sophocarpine by conversing Michael addition reaction in aqueous solution. Currently, WM130 (C_30_N_4_H_40_SO_5_F), a novel matrine derivative based on M19, was synthesized by replacing tertiary amine group with quaternary ammonium group (Figure 1). WM130 exhibited more stable chemical properties and better pharmacological activities than M19. In the present study, we intend to evaluate the antihepatic fibrosis activity of WM130 and elucidate its associated molecular mechanisms.

## 2. Materials and Methods

### 2.1. Cell Lines and Cell Culture

HSC-T6, an immortalized rat hepatic stellate cell line, was kindly gifted from Professor Weifen Xie (Department of Gastroenterology, Changzheng Hospital, Second Military Medical University, Shanghai, China). HSC-T6 cell line has been known to retain virtually all features of activated HSCs. The HSC-T6 cells of 12th passages were used in this research. Cells were maintained in Dulbecco's modified Eagle's medium (DMEM; Gibco, Gaithersburg, MD, USA) with 10% fetal bovine serum (FBS; Gibco) at 37°C, 5% CO_2_.

### 2.2. MTT Assay

HSC-T6 cells were seeded at a density of 5 × 10^3^ cells/well in 96-well plates. Eight h later, different compounds including WM1 series, WM2 series, WM3 series and the control drug M19 were added in culture at concentrations of 0.1 mg/mL. After incubation for 24 h at 37°C, twenty *μ*L of MTT (5 mg/mL) was added to each well. After a further incubation for 4 h, the supernatant was removed and 150 *μ*L of DMSO was added. Cell viability was measured at 492 nm. The experiment demonstrated that WM130 presents the highest potency among the tested compounds. Then the cells were exposed to different concentrations of WM130 (0, 17, 34, 51, 68, 85, 102, 119, 136, 153, and 170 *μ*M/well, which corresponded to 0, 10, 20, 30, 40, 50, 60, 70, 80, 90, and 100 *μ*g/well, resp.); four parallel wells were used for each concentration. M19 was installed as positive control. After incubation for 24 h at 37°C, cell viability was measured at 492 nm and IC_50_ was determined using the trimmed Spearman-Karber method [22].

### 2.3. BrdU Assay

HSC-T6 cells were seeded at a density of 1 × 10^4^ cells/well in 96-well plates. Cells were treated with 68 *μ*M (IC_50_) and 34 *μ*M (half IC_50_) of WM130 and 68 *μ*M of M19; phosphate-buffered saline (PBS) was used as blank control. After incubation for 24 h at 37°C, cells were pulsed with BrdU for an additional 8 h. Cell proliferation was measured by BrdU Cell Proliferation Assay kit (Merck Millipore, Germany) according to the manufacturer's instructions.

### 2.4. Scarification Test

HSC-T6 cells were seeded in six-well plates at a density of 2 × 10^5^ cell/well and maintained in DMEM with 10% FBS at 37°C and 5% CO_2_ for 8 h. Wounds were created in the cell monolayer by a pipette tip. The dead cells were washed away with 0.1 mM of PBS. Cells were treated with 68 *μ*M (IC_50_) and 34 *μ*M (half IC_50_) of WM130, 68 *μ*M of M19 in DMEM culture without FBS. The test was independently repeated for 3 times. Images were taken at 0 h, 12 h, 24 h, and 48 h. Migration distance was detected by Photoshop ver. 3.0.

### 2.5. Transwell Chamber Assay

HSC-T6 cells were seeded into the top chamber of 24-well transwell plates (8.0 *μ*m pore size; Corning, NY, USA) at a density of 5 × 10^5^ cell/well in 200 *μ*L serum-free DMEM media containing 2% FBS. Five hundred *μ*L DMEM media containing 20% FBS was added to the bottom chamber. After being cultured for 8 h, cells were incubated with 68 *μ*M (IC_50_) and 34 *μ*M (half IC_50_) of WM130 and 68 *μ*M of M19; PBS was used as blank control. The transwell plates were incubated at 37°C for 24 h and the migrated cells in the bottom chamber were stained by crystal violet and then quantified by microscope.

### 2.6. Hoechst Assay

HSC-T6 cells were seeded in six-well plates at a density of 5 × 10^3^ cell/well, incubated with 68 *μ*M (IC_50_) and 34 *μ*M (half IC_50_) of WM130 and 68 *μ*M of M19 for 24 h; PBS was used as blank control. The culture media were removed and the cells were fixed for 10 min with 0.5 mL of formaldehyde (4%) and then incubated with 0.5 mL Hoechst 33342 (Beyotime Biotechnology, Shanghai, China) for 10 min at room temperature and examined under a fluorescence microscope.

### 2.7. Flow Cytometry (FCM) to Examine Cell Apoptosis

HSC-T6 cells were seeded in six-well plates at a density of 1 × 10^5^ cell/well and incubated with 68 *μ*M (IC_50_) and 34 *μ*M (half IC_50_) of WM130 and 68 *μ*M of M19 for 24 h; PBS was used as blank control. Cells were stained with Annexin V-FITC and PI (KeyGEN BioTECH, Nanjing, China) and subsequently used in apoptosis evaluation with FCM.

### 2.8. RT-PCR Assay

HSC-T6 cells were seeded in six-well plates at a density of 2 × 10^5^ cell/well and incubated with 68 *μ*M (IC_50_) and 34 *μ*M (half IC_50_) of WM130 and 68 *μ*M of M19 for 24 h; PBS was used as blank control. Total RNA was extracted with Trizol (Life Technologies Corporation, New York, USA). Reverse transcription was performed with PrimeScript RT Reagent Kit (TaKaRa, Dalian, China). The cDNA was then amplified by polymerase chain reaction (PCR). The primers include the following: *β*-actin (537 bp, forward: 5′-ACC CAC ACT GTG CCC ATC TAT G-3′, reverse: 5′-AGA GTA CTT GCG CTC AGG AGG A-3′), *α*-SMA (120 bp, forward: 5′-CCG AGA TCT CAC CGA CTA CC-3′, reverse: 5′-TCC AGA GCG ACA TAG CAC AG- 3′), Collagen III (438 bp, forward: 5′-AGG CCA ATG GCA ATG TAA AG-3′, reverse: 5′-TAT TGG TGG GTG AA A CAG CA-3′), and TIMP-1 (250 bp, forward: 5′-TCC CCA GAA ATC ATC GAG AC-3′, reverse: 5′-TCA GAT TAT GCC AGG GAA CC-3′).

### 2.9. Western Blot

HSC-T6 cells were seeded in six-well plates at a density of 2 × 10^5^ cell/well and incubated with 68 *μ*M (IC_50_) and 34 *μ*M (half IC_50_) of WM130 and 68 *μ*M of M19 for 24 h; PBS was used as blank control. Cells were lysed in radio immunoprecipitation assay (RIPA) lysis buffer supplemented with phosphatase and protease inhibitors. Equal amounts of protein were separated by SDS-PAGE and transferred to PVDF membranes. The membranes were then blocked with 5% nonfat dry milk and incubated with the primary antibodies, including the rabbit antibodies to rat AKT, p-AKT, ERK, p-ERK, EGFR, p-EGFR, and p-Raf (Cell Signaling Technology, Danvers, MA), the mouse antibodies to rat TGF-*β*1, *α*-SMA (Abcam Inc. Cambridge, MA) and p-Smad (Santa Cruz Biotech Inc., CA), and the glyceraldehyde 3-phosphate dehydrogenase (GAPDH) antibody (Kangchen Bio-tech, Shanghai, China), followed by incubation with the secondary antibodies, horseradish peroxidase-conjugated sheep anti-mouse immunoglobulin (Ig) G or sheep anti-rabbit IgG (Abcam Inc. Cambridge, MA). Blots were developed using an enhanced chemiluminescence detection kit (Beyotime Biotechnology, Shanghai, China). The agarose gel bands were scanned for gray density analysis relative to the loading control by ImageJ software.

### 2.10. ELISA Determination of Collagen I

HSC-T6 cells were seeded in six-well plates at a density of 2 × 10^5^ cell/well and incubated with 68 *μ*M (IC_50_) and 34 *μ*M (half IC_50_) of WM130 and 68 *μ*M of M19 for 24 h; PBS was used as blank control. The expression levels of Collagen I in the culture media were determined by ELISA with a commercial ELISA kit (Westang Bio-Tech, Shanghai, China) following the manufacturer's instruction.

### 2.11. Animal Model Experiment

Twenty-five male Sprague Dawley (SD) rats, initially weighing 80–100 g, were purchased from the Shanghai Laboratory Animal Center (Shanghai, China). All animals were allowed free access to rodent feed and tap water. All efforts were made to minimize suffering of the animals and all animal studies performed were in accordance with the standard approved by the Animal Care Committee of Second Military Medical University. Rats were randomly allocated to five groups: the normal control group, DMN group, M19 68 *μ*M/kg + DMN group, WM130 34 *μ*M/kg + DMN group, and WM130 68 *μ*M/kg + DMN group, *n* = 5 in each group. The model of liver fibrosis was induced by intraperitoneal injection with 1% DMN (10 *μ*g/kg body weight) for three consecutive days per week for up to four weeks. Then rats in the treatment groups were administered orally by gavage with M19 or WM130 three times per week for 4 weeks. After treatments, the rats were maintained and observed for two months. At the end of the experiment, rats were all sacrificed by anesthetization. Liver and serum samples were collected. Four *μ*m thick liver sections were processed by Van Gieson's (V.G) staining (Fuzhou Maixin Biotech. Co., Ltd., Fuzhou, China) and were used to assess the hepatic collagen deposition and the expression levels of TGF-*β*1, p-AKT, *α*-SMA, and p-ERK with immunohistochemistry. Serum levels of alanine transaminase (ALT) and aspartate transaminase (AST) were detected with automatic biochemical analyser. The contents of Collagen I in rat sera were determined with ELISA.

### 2.12. Statistical Analysis

The results were reported as mean ± SD. Statistical analysis was performed and differences between control and other groups were tested by one-way ANOVA with SPSS 14.0 statistical package. *P* < 0.05 was considered statistically significant.

## 3. Results

### 3.1. WM130 Inhibits the Proliferation of HSC-T6 Cells

**Figure 2:**
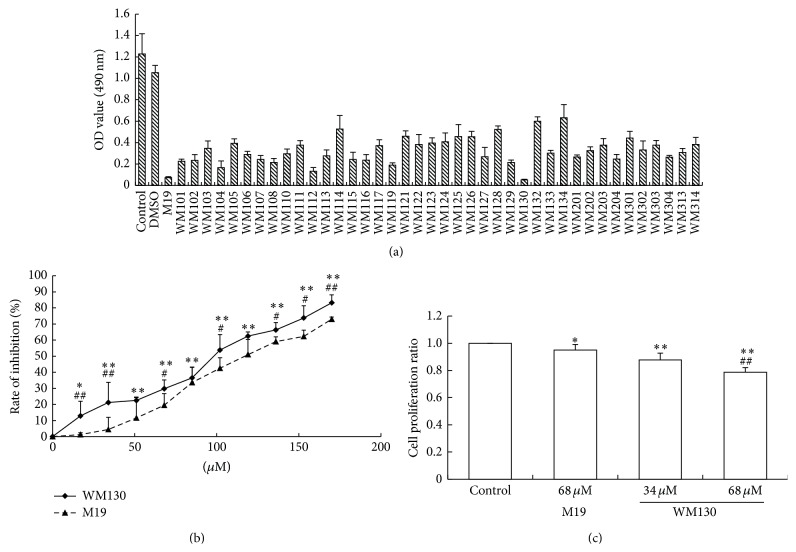
WM130 and M19 inhibited the proliferation of HSC-T6 cells* in vitro*. (a) HSC-T6 cells were treated for 24 h with a series of different compounds at concentration of 0.1 mg/mL, and the cell viability was measured by MTT assay. (b) The effect of a series of different concentrations of WM130 or M19 was tested on cell viability of HSC-T6 cells. The inhibition rates were calculated by [inhibition rate = (1 − OD value of treatment group)/OD value of control group]. ^*∗*^ *p* < 0.05, ^*∗∗*^ *p* < 0.01 versus control group; ^#^ *p* < 0.05, ^##^ *p* < 0.01 versus M19 group.

The OD values of HSC-T6 cells treated with a series of matrine derivatives were showed in Figure 2(a), of which WM130 exhibited better bioactivity. Compared with the parental HSC-T6 cells without treatment, both WM130 and M19 dose-dependently inhibited the cell viability* in vitro*, and the inhibition effect of WM130 was higher than that of M19 (Figure 2(b)). The IC_50_ of WM130 and M19 was 68 *μ*M and 106 *μ*M, respectively. The similar results were obtained by BrdU assay that was performed to test the effect of WM130 and M19 on proliferation of HSC-T6 cells (Figure 2(c)). WM130 could inhibit proliferation of HSC-T6 cells, and lower cell proliferation was not due to the induction of cell apoptosis.

### 3.2. WM130 Inhibits the Migration and Invasion of HSC-T6 Cells

**Figure 3:**
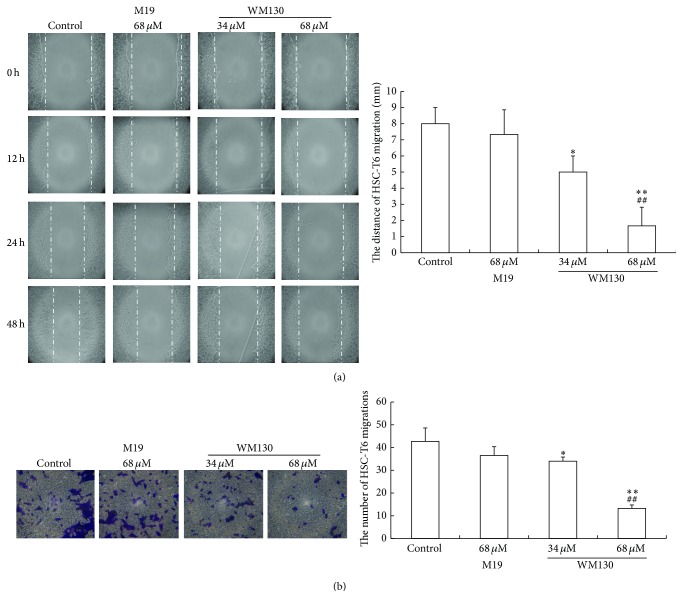
WM130 inhibited the migration and invasion of HSC-T6 cells. (a) The migration ability of HSC-T6 cells was analyzed by a scratch motility assay. WM130 68 *μ*M group revealed a less migration distance during the treatment period of  0–48 h, compared with the control group or M19 group. (b) The effect of WM130 on inhibiting invasion of HSC-T6 cells was examined by transwell chamber assay. Invasive ability of HSC-T6 cells was quantified by counting the number of stained cells under microscopy (at 200x magnification). ^*∗*^ *p* < 0.05, ^*∗∗*^ *p* < 0.01 versus Control group; ^##^ *p* < 0.01 versus M19 68 *μ*M group.

HSC-T6 cell motility was observed by wound healing assay to investigate the function of WM130. Twenty-four h after scratching, HSC-T6 cells treated with WM130 or M19 had healed the wound to a less extent than the cells in the control group. The results indicated that the migration activity of HSC-T6 cells treated with WM130 was markedly inhibited (Figure 3(a)).

The effect of WM130 on HSC-T6 cell invasion was evaluated by transwell chamber experiment. Consistent with the migration results, HSC-T6 cells treated with WM130 or M19 exhibited lower invasion ability from the top of the chamber to the bottom during a 24 h treatment. Compared with the control group, the numbers of invasive cells in the WM130 34 *μ*M and 68 *μ*M groups were 34.00 ± 1.83 (*p* < 0.05) and 13.25 ± 1.50 (*p* < 0.01), respectively, while they were 36.50 ± 3.87 (*p* > 0.05) in the M19 68 *μ*M group. The invasion assay demonstrated that WM130 inhibited the invasive ability of HSC-T6 cells than M19 (Figure 3(b)).

### 3.3. WM130 Induces Apoptosis of HSC-T6 Cells

**Figure 4:**
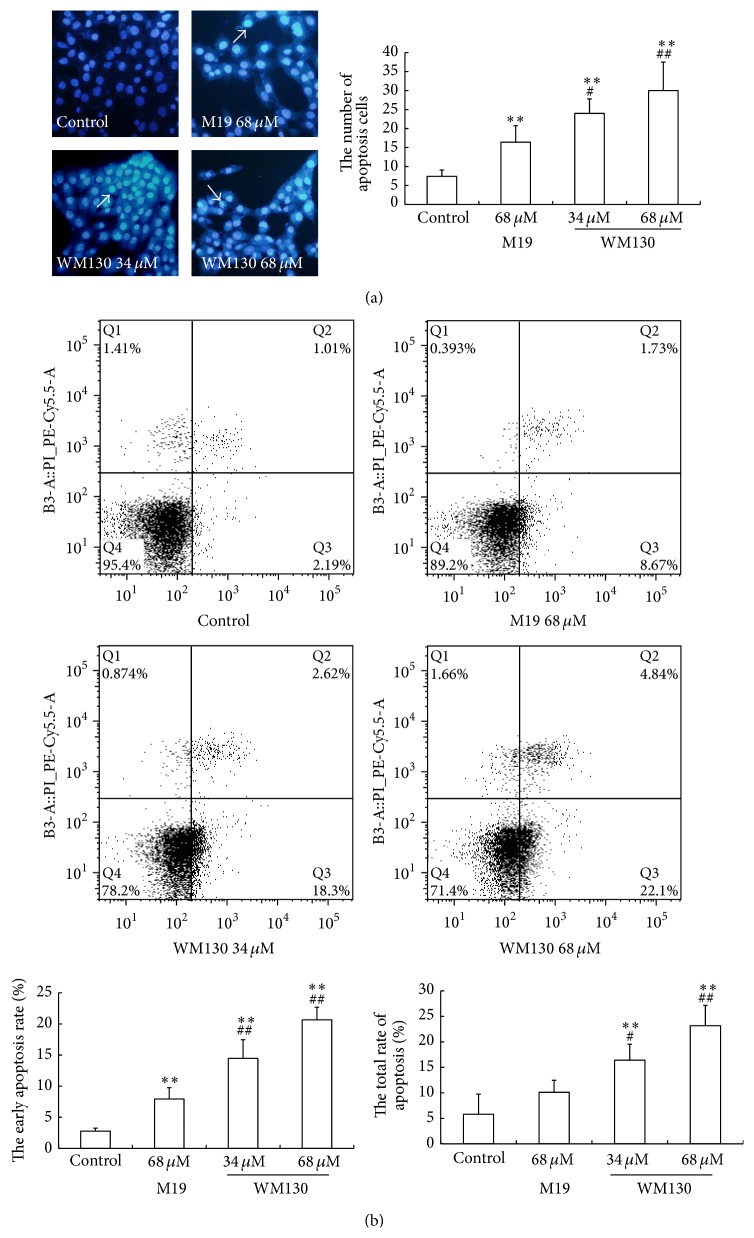
WM130 induces apoptosis of HSC-T6 cells. (a) Blue stained nuclei were dyed by Hoechst and analyzed under fluorescent microscope (at 200x magnification). Apoptotic body could be observed in WM130 68 *μ*M group (white arrow). More apoptotic HSC-T6 cells could be detected in WM130 68 *μ*M group than in other treated group. (b) The Annexin V/PI apoptosis kit was used to quantify the percentage of cells undergoing apoptosis. The early apoptotic rates and the total apoptotic rates of HSC-T6 cells were detected in different groups by flow cytometry. ^*∗∗*^ *p* < 0.01 versus control group; ^#^ *p* < 0.05, ^##^ *p* < 0.01 versus M19 68 *μ*M group.

Cellular nuclear shrinkage, apoptotic body formation, nuclear condensation, and DNA fragmentation are the hallmarks of apoptosis [23]. Hoechst staining showed that nuclei of cells in the control group showed weak blue homogeneous staining. Distinct chromatin condensation was identified in the treatment groups. The nuclear fragmentation was observed in the WM130 68 *μ*M group. With the dose of WM130 increasing, the numbers of apoptotic HSC-T6 cells were increased (Figure 4(a)). The annexin V/PI apoptosis kit was used to quantify the percentage of cells undergoing apoptosis. As shown in Figure 4(b), the apoptosis of HSC-T6 cells was 23.18 ± 4.00% and 10.10 ± 2.36% in response to treatment with 68 *μ*M of WM130 and 68 *μ*M of M19, respectively, and the early apoptosis of HSC-T6 cells was 20.65 ± 2.04% and 7.94 ± 1.82% in response to treatment with 68 *μ*M of WM130 and 68 *μ*M of M19, respectively. Therefore, WM130 exhibited a stronger effect on inducing apoptosis of HSC-T6 cells than M19.

### 3.4. WM130 Inhibits HSC-T6 Cell Activation and ECM Production

**Figure 5:**
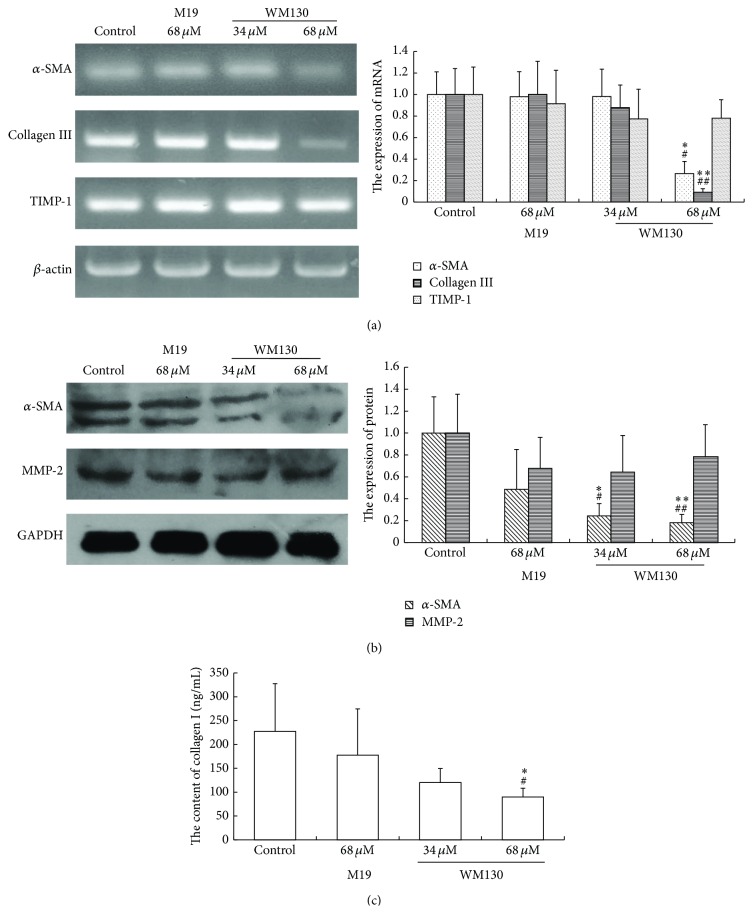
WM130 inhibits the activation of HSC-T6 cells and the secretion of ECM. (a) All samples were collected after incubation with WM130 and M19 for 24 h. Total RNA was extracted and the mRNA expression levels of *α*-SMA, Collagen I, and Collagen III in different groups were examined by RT-PCR assay. (b) Cells were lysed in RIPA lysis buffer supplemented with PMSF and the protein expression levels of *α*-SMA and MMP-2 in different groups were quantified by Western Blot assay. (c) Cell culture media in different groups were collected in which the concentrations of collagen I were measured by ELISA assay. All experiments were independently performed 3 times. ^*∗*^ *p* < 0.05, ^*∗∗*^ *p* < 0.01 versus control group; ^#^ *p* < 0.05, ^##^ *p* < 0.01 versus M19 68 *μ*M group.

Activated HSCs can express *α*-SMA and secret ECM. Previous studies demonstrated that the proteolytic activity of collagen was regulated by matrix metalloproteinases (MMPs), which were in turn regulated by their endogenous inhibitors, tissue inhibitor of metalloproteinases (TIMPs). By RT-PCR and Western Blot analyses, we observed WM130 or M19 reduced the mRNA and protein levels of *α*-SMA, Collagen I, and Collagen III in HSC-T6 cells. However, there were no significant differences between the levels of MMP-2, as well as TIMP-1, in the treated groups and the control group (Figure 5). These data indicated that WM130 performed more effectively than M19 in inhibiting the activation of HSC-T6 cells and the production of ECM.

### 3.5. WM130 Inhibits TGF-*β*/Smad and Ras/ERK Pathways

**Figure 6:**
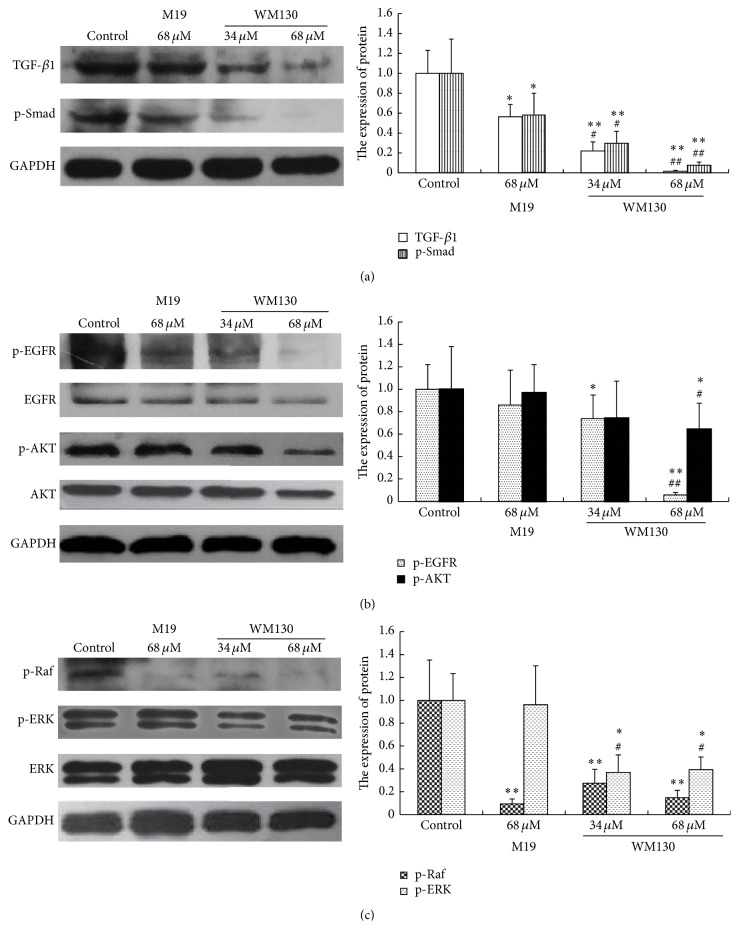
WM130 inhibits TGF-*β*/Smad and Ras/ERK pathways. All samples were collected after incubation with WM130 and M19 for 24 h. Total protein was extracted by RIPA and the expression levels of (a) TGF-*β*1, p-Smad, (b) p-EGFR, p-AKT, and (c) p-Raf, p-ERK in HSC-T6 cells in different treated groups were determined by Western Blot assay. The experiments were independently performed 3 times. ^*∗*^ *p* < 0.05, ^*∗∗*^ *p* < 0.01 versus control group; ^#^ *p* < 0.05, ^##^ *p* < 0.01 versus M19 68 *μ*M group.

TGF-*β*/Smad and Ras/ERK signal pathways are closely associated with liver fibrosis. TGF-*β*1 is a major profibrogenic cytokine associated with organ fibrogenesis. The expression levels of TGF-*β*1, p-Smad, p-EGFR, p-AKT, p-Raf, and p-ERK were determined by Western Blot analysis. The results revealed that the expression levels of TGF-*β*1, p-Smad, p-EGFR, p-AKT, p-Raf, and p-ERK were obviously downregulated in WM130- and M19-treated groups (Figure 6), and WM130 is a more effective compound in inhibiting the activated TGF-*β*/Smad and Ras/ERK pathways in HSC-T6 cells than M19.

### 3.6. WM130 Inhibits DMN-Induced Rat Liver Fibrosis

**Figure 7:**
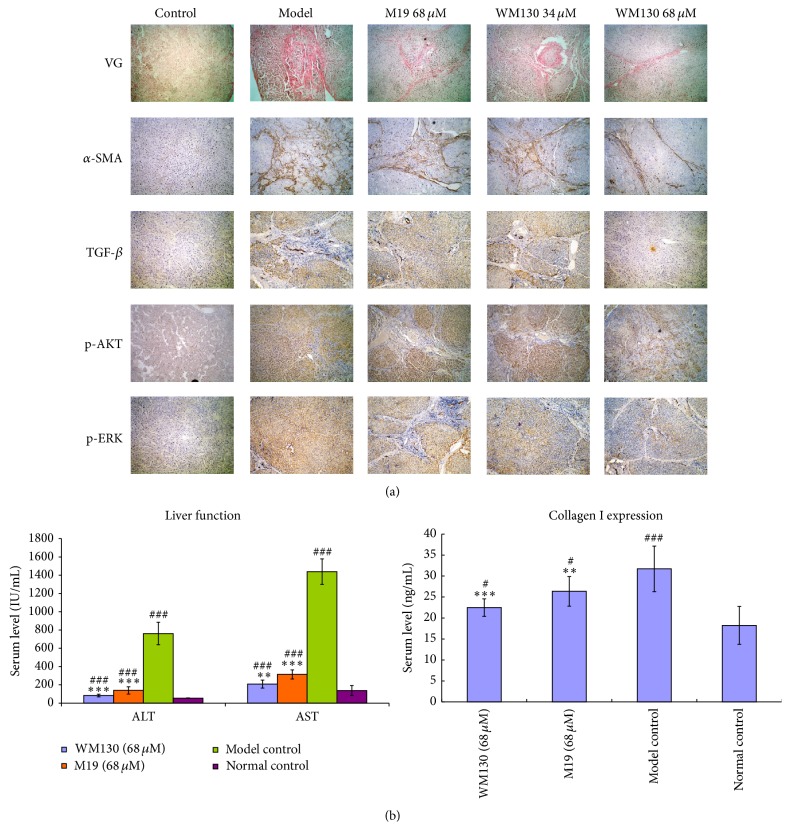
WM130 inhibits DMN-induced rat liver fibrosis. The model of liver fibrosis was induced by intraperitoneal injection with 1% DMN. Then rats were treated with M19 or WM130 by gavage. (a) The contents of the collagen fiber in liver tissue of DMN-induced rat were detected by Van Gieson's staining. The immunohistochemical staining was used to observe *α*-SMA, TGF-*β*1, p-AKT, and p-ERK positive areas in liver tissues of DMN-injured rats (at 200x magnification). (b) Sera were prepared from rat blood and used to determine the contents of ALT, AST, and Collagen I. ^*∗∗*^ *p* < 0.01, ^*∗∗∗*^ *p* < 0.001 versus normal Control; ^#^ *p* < 0.05, ^###^ *p* < 0.001 versus model Control.

Repeat injections of DMN in rats cause liver fibrosis. Collagen fiber could be detected by Van Gieson's Staining. In the liver fibrosis rats, significant proliferation of collagen fiber (red staining) was observed. Collagen fiber was downregulated after treatment with WM130 or M19. Compared with the control normal group, the expression levels of *α*-SMA were obviously increased in DMN-induced fibrotic rats, which were attenuated by WM130 or M19 treatments. In addition, immunohistochemical analyses were conducted to measure DMN-induced liver fibrosis. The expression of TGF-*β*1, p-AKT, and p-ERK was all upregulated in DMN-induced rats, and WM130 or M19 could downregulate the levels of these proteins (Figure 7). The results suggested that WM130 and M19 could inhibit liver fibrosis in DMN-induced rats, and WM130 performed more effectively than M19 in antihepatic fibrosis activity, which was consistent with the* in vitro* results. Sera were prepared from rat blood and used to determine the contents of ALT, AST, and Collagen I. WM130 and M19 treatments resulted in an obvious decrease of serum ALT, AST, and Collagen I compared with the DMN-induced model control group, but they were also higher than that in the normal control mice (Figure 7(b)).

## 4. Discussion

Liver fibrosis frequently resulted from liver chronic damage can lead to development of liver cirrhosis and consequent failure of hepatic function. During the process of fibrogenesis, the proliferation and activation of HSCs are believed to play essential roles [24, 25]. Therefore, suppression or reversal of HSC activation is considered as the main target of hepatic fibrosis. In this study, the immortalized HSC-T6 cell line was used in the* in vitro* experiments. These cells have been known to retain virtually all features of activated HSCs and showed *α*-SMA positive expression (Supplementary Figure 1 in Supplementary Material available online at http://dx.doi.org/10.1155/2015/203978).

Matrine, an active ingredient of* Sophora flavescens* (*S. flavescens* Ait), is widely used in clinic to treat chronic liver disease. This substance has been known for its pharmacological effects including anti-inflammatory, immunosuppressive, antitumor, and anti-liver fibrosis. However, the potency of matrine is low. We reconstructed Sophocarpine, whose structure was similar to matrine, with thiosulfate and Michael addition, to generate a series of matrine derivates. M19 is one of thiosulfate sophocarpine derivates with high bioactivity and low side effect [20], but it can easily turn back into thiosulfate sophocarpine by reversing Michael addition reaction in aqueous solution. Then we reconstructed the structure of M19, with 18-methylamino-acylated to improve its stability, with amino side chain added to enhance its activity. Finally, we got WM130 maleate, a novel matrine derivative, with more stable chemical properties and better pharmacological activities. Our experiments showed that HSC-T6 was more sensitive in response to WM130. In the present study, rats were maintained and observed for two months after treatment with WM130. We estimated the therapeutic functions, as well as any long-term side effect and toxicity of WM130 by a long observation period. We could not find any significant liver toxicity of WM130* in vivo*. The toxicity assay of WM130 was determined on normal liver cells WRL-68; the IC_50_ of WM130 in WRL-68 cells was 116.4 *μ*M (Supplementary Figure 2), which was much higher than the doses of 68 *μ*M and 34 *μ*M used in the* in vitro* experiments. The results demonstrated that WM130 has no side toxicity at the concentration of 34 or 68 *μ*M* in vivo* and* in vitro*. This experiment intended to study the inhibitory effect of WM130 on HSC activation and liver fibrosis in rats and investigated the possible molecular mechanism, hoping to provide an aggressive treatment for hepatic fibrosis.

We investigated the effect of WM130 and M19 on the proliferation of HSC-T6 by MTT and BrdU assays. HSC-T6 cell proliferation could be inhibited by WM130 or M19, and the IC_50_ values of WM130 and M19 were 68 *μ*M and 106 *μ*M, respectively. So we selected the doses of 68 *μ*M (IC_50_) and 34 *μ*M (half of IC_50_) of WM130 and 68 *μ*M of M19 in the cytological experiments. By using Scarification test and Transwell chamber assay, we found that WM130 could attenuate the migratory and invasive capacities of HSC-T6 cells. With hoechst staining and FCM assay, we provided evidence that WM130 induced more significant apoptosis when compared with M19. In addition, the WM130 68 *μ*M group induced a greater degree of apoptosis than the M19 68 *μ*M group. Therefore, WM130 and M19 both could efficiently inhibit proliferation, migratory, and invasion as well as induce apoptosis in HSC-T6 cells. Moreover, WM130 preformed more effectively in suppressing the activation of HSCs than M19* in vitro*.

It was confirmed that the activated HSCs are the principal *α*-SMA-producing cells and the major source of ECM, such as Collagen I, Collagen III, and proteoglycans, in liver fibrosis. MMPs and TIMPs play an important part in ECM remodeling. MMPs, a family of zinc metalloendopeptidase, have ability to degrade the protein components of ECM. TIMPs were inhibitors of MMPs, of which TIMP-1 was highly expressed in liver [26]. In our study, the expressions of *α*-SMA, Collagen I, and Collagen III were all downregulated by WM130 and M19 in HSC-T6 cells* in vitro*. In addition, collagen fiber was significantly increased after repeat injections of DMN, and they all could be downregulated by treatment of M19 or WM130. The increased expression of *α*-SMA in DMN-injured rat livers was attenuated by WM130 and M19, and WM130 performed more effectively than M19. However, the expression of MMP-2 and TIMP-1 had no significant changes in different treated groups neither in the* in vitro* nor in the* in vivo* experiments. Therefore, we can draw the conclusion that WM130 can inhibit the activation of HSC-T6 cells and the deposition of ECM, and there is no correlation between the balances of MMP-2/TIMP-1 with the deposition of ECM.

TGF-*β*1 is the most potent fibrogenic cytokine in the liver. The stimulation of activated HSCs by TGF-*β*1 is believed to be a crucial fibrogenic response in liver fibrosis, because the high expression of TGF-*β*1 in the activated HSCs can lead to increase of ECM [27]. In addition, TGF-*β*/Smad signaling is the main pathway which could be regulated in liver fibrosis [28]. It has demonstrated that TGF-*β*1 was capable of binding to its receptor, which could lead to phosphorylation of Smad. AKT can be activated by p-Smad through PI3K/AKT pathway [29, 30], involved in the progress of hepatic fibrosis. Furthermore, the study revealed that EGFR, binding with EGF and TGF-*β*1, can specifically promote cell proliferation and differentiation [31]. We observed that the expression of TGF-*β*1 and p-AKT was upregulated in liver tissue of DMN-induced rats, which was attenuated by WM130 and M19, and WM130 performed more effectively than M19. The results of the* in vitro* experiments also showed that the expression of TGF-*β*1, p-Smad, p-AKT, and p-EGFR in HSC-T6 cells was all suppressed by WM130. These results clearly indicated that WM130 can inhibit the hepatic fibrosis by blocking the activation of TGF-*β*/Smad pathway.

MAPK had been implicated in the regulation of diverse cellular processes, including embryogenesis, proliferation, differentiation, and apoptosis [32]. After HSC activation, the released stimulators activated Ras and promoted the proliferation and differentiation of HSCs through Ras-MEK-ERK pathway [33]. Consistently, we observed an increased level of p-ERK in liver tissues of DMN-induced rats, which can be decreased by WM130 or M19 treatments. Moreover, the expression of p-Raf and p-ERK in WM130 or M19-treated HSC-T6 cells was suppressed, and the effects of WM130 were better than M19. These results suggested that WM130 can suppress hepatic fibrosis through regulating the Ras/ERK pathway, and WM130 exhibited a better antihepatic fibrosis activity than M19.

## 5. Conclusions

We have presented strong experimental evidence that WM130, a novel matrine derivative, could suppress the activation of HSCs and hepatic fibrosis induced both in cytological experiments and in the DMN-induced rat model by inducing apoptosis and suppressing deposition of ECM. The molecular mechanism of WM130 may possibly involve multiple targets, such as blocking TGF-*β*/Smad and Ras/ERK pathways. Thus, we proposed that WM130 could be further developed as a new drug candidate for treatment of hepatic fibrosis.

## Supplementary Material

The purity of HSC-T6 cells were identified by α-SMA staining. All the HSC-T6 cells showed positive staining (Supplementary Figure 1). The result demonstrated that the HSC-T6 cell we used in this research was pure.
The toxicity assay of WM130 was determined on normal liver cells WRL-68; the IC50 of WM130 in WRL-68 cells was 116.4 µM (Supplementary Figure 2), which was much higher than the doses of 68 µM and 34 µM used in the in vitro experiments. The results demonstrated that WM130 has no side toxicity at the concentration of 34 or 68 µM in vivo and in vitro.

## References

[1] Sakata K., Eda S., Lee E.-S., Hara M., Imoto M., Kojima S. (2014). Neovessel formation promotes liver fibrosis via providing latent transforming growth factor-*β*. *Biochemical and Biophysical Research Communications*.

[2] Hu L., Peng X., Tang Y., Liu Y. (2013). Synthesis of peptides of Carapax Trionycis and their inhibitory effects on TGF-*β*1-induced hepatic stellate cells. *Drug Discoveries & Therapeutics*.

[3] Borg B. B., Seetharam A., Subramanian V. (2011). Immune response to extracellular matrix collagen in chronic hepatitis C-induced liver fibrosis. *Liver Transplantation*.

[4] Dooley S., Delvoux B., Lahme B., Mangasser-Stephan K., Gressner A. M. (2000). Modulation of transforming growth factor *β* response and signaling during transdifferentiation of rat hepatic stellate cells to myofibroblasts. *Hepatology*.

[5] Nakao A., Afrakhte M., Morén A. (1997). Identification of Smad7, a TGF*β*-inducible antagonist of TGF-*β* signalling. *Nature*.

[6] Tahashi Y., Matsuzaki K., Date M. (2002). Differential regulation of TGF-beta signal in hepatic stellate cells between acute and chronic rat liver injury. *Hepatology*.

[7] Sancho-Bru P., Juez E., Moreno M. (2010). Hepatocarcinoma cells stimulate the growth, migration and expression of pro-angiogenic genes in human hepatic stellate cells. *Liver International*.

[8] Park S.-J., Sohn H.-Y., Yoon J., Park S. I. (2009). Down-regulation of FoxO-dependent c-FLIP expression mediates TRAIL-induced apoptosis in activated hepatic stellate cells. *Cellular Signalling*.

[9] Ji L., Xue R., Tang W. (2014). Toll like receptor 2 knock-out attenuates carbon tetrachloride (CCl_4_)-induced liver fibrosis by downregulating MAPK and NF-*κ*B signaling pathways. *FEBS Letters*.

[10] Chen X. G., Xu C. S., Liu Y. M. (2013). Involvement of ERK1/2 signaling in proliferation of eight liver cell types during hepatic regeneration in rats. *Genetics and Molecular Research*.

[11] Bao L. L., Yan Y., Xu C. (2013). MicroRNA-21 suppresses PTEN and hSulf-1 expression and promotes hepatocellular carcinoma progression through AKT/ERK pathways. *Cancer Letters*.

[12] Gressner A. M. (1996). Transdifferentiation of hepatic stellate cells (Ito cells) to myofibroblasts: a key event in hepatic fibrogenesis. *Kidney International, Supplement*.

[13] Zhang B., Liu Z.-Y., Li Y.-Y. (2011). Antiinflammatory effects of matrine in LPS-induced acute lung injury in mice. *European Journal of Pharmaceutical Sciences*.

[14] Yu J. L., Li J. H., Chengz R. G., Ma Y. M., Wang X. J., Liu J. (2014). Effect of matrine on transforming growth factor *β*1 and hepatocyte growth factor in rat liver fibrosis model. *Asian Pacific Journal of Tropical Medicine*.

[15] Liu Y., Xu Y., Ji W. D. (2014). Anti-tumor activities of matrine and oxymatrine: literature review. *Tumor Biology*.

[16] Wang Y., Yuan J., Yuan X. (2012). Observation of antinociceptive effects of Oxymatrine and its effect on delayed rectifier K^+^ currents (Ik) in PC12 cells. *Neurochemical Research*.

[17] Hu Z.-L., Zhang J.-P., Yu X.-B., Lin W., Qian D.-H., Wan M.-B. (1996). Effect of matrine on lipopolysaccharides/D-galactosamine-induced hepatitis and tumor necrosis factor release from macrophages *in vitro*. *Acta Pharmacologica Sinica*.

[18] Zhang J. P., Zhang M., Zhou J. P. (2001). Antifibrotic effects of matrine on *in vitro* and *in vivo* models of liver fibrosis in rats. *Acta Pharmacologica Sinica*.

[19] Yang J., Hou Y., Ji G. (2014). Targeted delivery of the RGD-labeled biodegradable polymersomes loaded with the hydrophilic drug oxymatrine on cultured hepatic stellate cells and liver fibrosis in rats. *European Journal of Pharmaceutical Sciences*.

[20] Hu H., Wang S., Zhang C. (2010). Synthesis and *in vitro* inhibitory activity of matrine derivatives towards pro-inflammatory cytokines. *Bioorganic and Medicinal Chemistry Letters*.

[21] Xu W. H., Hu H. G., Tian Y. (2014). Bioactive compound reveals a novel function for ribosomal protein S5 in hepatic stellate cell activation and hepatic fibrosis. *Hepatology*.

[22] Hamilton M. A., Russo R. C., Thurston R. V. (1977). Trimmed Spearman-Karber method for estimating median lethal concentrations in toxicity bioassays. *Environmental Science & Technology*.

[23] Wang H. B., Ma X. Q. (2014). *β*, *β*-dimethylacrylshikonin induces mitochondria-dependent apoptosis of human lung adenocarcinoma cells in vitro via p38 pathway activation. *Acta Pharmacologica Sinica*.

[24] Kim K. S., Yang H. J., Lee J. Y. (2014). Effects of *β*-sitosterol derived from *Artemisia capillaris* on the activated human hepatic stellate cells and dimethylnitrosamine-induced mouse liver fibrosis. *BMC Complementary and Alternative Medicine*.

[25] Yu F. X., Teng Y. Y., Zhu Q. D., Zhang Q. Y., Tang Y. H. (2014). Inhibitory effects of capsaicin on hepatic stellate cells and liver fibrosis. *Biochemistry and Cell Biology*.

[26] Rockey D. C. (1999). Gene therapy for hepatic fibrosis-bringing treatment into the new millennium. *Hepatology*.

[27] Rocha S. W., de França M. E., Rodrigues G. B. (2014). Diethylcarbamazine reduces chronic inflammation and fibrosis in carbon tetrachloride- (CCl_4_-) induced liver injury in mice. *Mediators of Inflammation*.

[28] Lee J. H., Jang E. J., Seo H. L. (2014). Sauchinone attenuates liver fibrosis and hepatic stellate cell activation through TGF-*β*/Smad signaling pathway. *Chemico-Biological Interactions C*.

[29] Zhao B., Chen Y. G. (2014). Regulation of TGF-*β* signal transduction. *Scientifica*.

[30] Park S. H., Cho H. J., Jeong Y. J. (2014). Melittin inhibits TGF-*β*-induced pro-fibrotic gene expression through the suppression of the TGF*β*RII-Smad, ERK1/2 and JNK-mediated signaling pathway. *The American Journal of Chinese Medicine*.

[31] Voon D. C.-C., Wang H., Koo J. K. W. (2013). EMT-induced stemness and tumorigenicity are fueled by the EGFR/Ras pathway. *PLoS ONE*.

[32] Lawrence M. C., Jivan A., Shao C. (2008). The roles of MAPKs in disease. *Cell Research*.

[33] Wang H., Guan W., Yang W. (2014). Caffeine inhibits the activation of hepatic stellate cells induced by acetaldehyde via adenosine A2A receptor mediated by the cAMP/PKA/SRC/ERK1/2/P38 MAPK signal pathway. *PLoS ONE*.

