# Supplementary material for: A Novel Matrine Derivative WM130 Inhibits Activation of Hepatic Stellate Cells and Attenuates Dimethylnitrosamine-Induced Liver Fibrosis in Rats
Source: Biomed Res Int. 2015 Jun 18;2015:203978. doi: 10.1155/2015/203978 (PMC4488526; doi:10.1155/2015/203978)

**Supplement Figure Legends**

Supplementary Figure 1: Purity identification of HSC-T6 cells. HSC-T6 cells were seeded at a density of $1\times10^4$ cells/well in 6-well plates. After incubation for 24 h at 37 ℃, α-SMA in cells were examined by immunohistochemical method. All the cells showed positive staining.

Supplementary Figure 2: Toxicity assay of WM130 in WRL-68 cells. normal liver cells WRL-68 were seeded at a density of $5\times10^3$ cells/well in 96-well plates. Different compounds were added in culture at equal concentrations (0.1 mg/ml). After incubation for 24 h, MTT assay were obversed. The $IC_{50}$ of WM130 was 116.4 μM.

**Supplementary Figure 1**

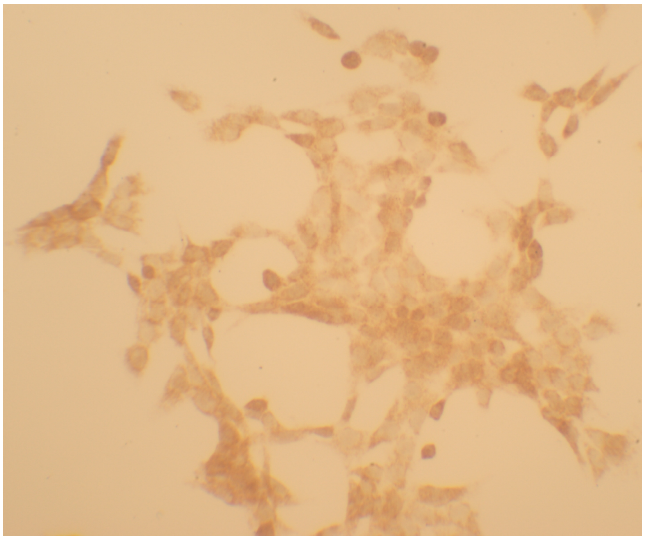

**Supplementary Figure 2**

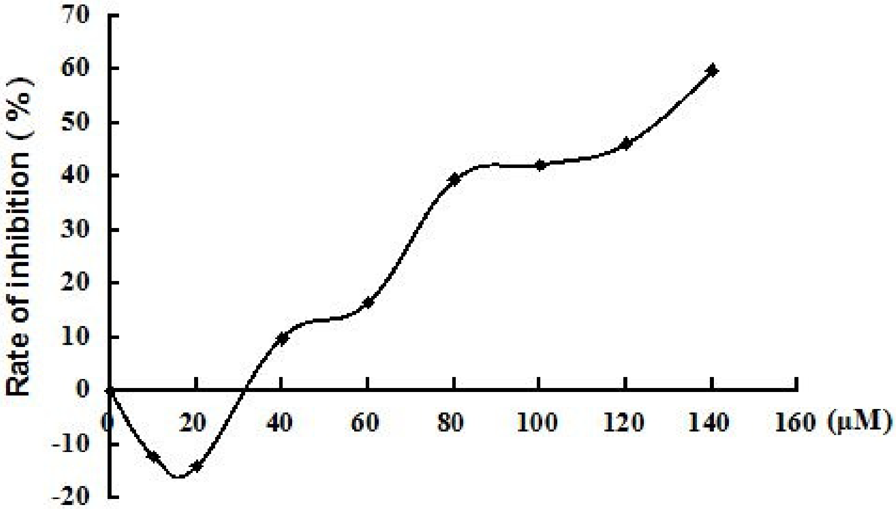

Supplement: Supplementary file 1 — The purity of HSC-T6 cells were identified by α-SMA staining. All the HSC-T6 cells showed positive staining (Supplementary Figure 1). The result demonstrated that the HSC-T6 cell we used in this research was pure. The toxicity assay of WM130 was determined on normal liver cells WRL-68; the IC50 of WM130 in WRL-68 cells was 116.4 µM (Supplementary Figure 2), which was much higher than the doses of 68 µM and 34 µM used in the in vitro experiments. The results demonstrated that WM130 has no side toxicity at the concentration of 34 or 68 µM in vivo and in vitro. [file 203978.f1.pdf]
